# Impact of 3D Printing Technique and TPE Material on the Endurance of Pneumatic Linear Peristaltic Actuators

**DOI:** 10.3390/mi13030392

**Published:** 2022-02-28

**Authors:** Miranda Fateri, João Falcão Carneiro, Constantin Schuler, João Bravo Pinto, Fernando Gomes de Almeida, Udo Grabmeier, Tobias Walcher, Michael Salinas

**Affiliations:** 1 Faculty Mechanical Engineering & Materials Science, Aalen University, Beethovenstraße 1, 73430 Aalen, Germany; constantin.schuler@studmail.htw-aalen.de (C.S.); udo.grabmeier@hs-aalen.de (U.G.); tobias.walcher@hs-aalen.de (T.W.); 2 LAETA-INEGI, Faculty of Engineering, University of Porto, Rua Dr. Roberto Frias, s/n, 4200-465 Porto, Portugal; jpbrfc@fe.up.pt (J.F.C.); jpp.professional@gmail.com (F.G.d.A.); 3 LAETA-INEGI, Department of Mechanical Engineering, University of Porto, Rua Dr. Roberto Frias, s/n, 4200-465 Porto, Portugal; jbpinto@inegi.up.pt; 4 ARBURG GmbH + Co KG, Penzendorfer Straße 10, 91126 Rednitzhembach, Germany; michael_salinas@arburg.com

**Keywords:** linear peristaltic actuators, pneumatic actuators, additive manufacturing, 3D printing, thermoplastic elastomers

## Abstract

In this paper, additive manufacturing was used in order to produce hose prototypes for peristaltic linear pneumatic actuators. In order to optimise the endurance of the actuator, we 3D printed different thermoplastic polyurethane elastomers with different shore hardness levels using ARBURG Plastic Freeforming technology. Furthermore, effects of the hose geometries on the lifetime of the actuator were investigated. Experimental evidence showed that the lifetime of the actuator was dependent on the combination of the hose design and on the material used to manufacture the hose. Moreover, experimental tests showed that the use of the Aurburg-Freeformer 3D printing technology led to a much higher hose endurance than the one reported by using the fused layer manufacturing technique.

## 1. Introduction

Interest in development of interactive robots capable of co-working and interacting with humans is gaining significant attention from different industries in recent years. Following this, pneumatic actuated systems could play a significant role in this context, as they are a simple solution to ensure safety requirements in cases of human–robot contact and offer motion capabilities which are not achievable using more rigid actuation solutions [1,2,3]. In fact, co-working robots based on electrical actuators require complex control strategies to reduce their natural high stiffness. On the contrary, pneumatic actuators have an inherent high compliance, and thus safety in the event of contact with the environment is naturally obtained. These reasons have been pushing the scientific community to explore new ways to implement pneumatic actuators. Several approaches have been followed, namely, by (i) using flexible materials [4,5,6,7,8,9,10] to embody the actuator, (ii) using hybrid electric-pneumatic actuators that combine the simpleness of control of electrical actuators with the pneumatic actuators low stiffness [11,12], and (iii) developing a new piston configuration to increase the energetic efficiency of conventional cylinders [13].

Among the recent reported actuation systems, peristaltic linear pneumatic actuators (PLPA) have been concluded to be a simple, cost-efficient alternative to other actuators [14,15,16], while presenting significant advantages for servo control, namely, beneficial friction characteristics for that purpose.

PLPA actuators are driven by pneumatic energy. They function on the basis of a moving body composed by two rollers that press a hose until it becomes sealed in the middle, creating two separate isolated chambers (see [17]). When air is pumped into one of the chambers, rollers will move in the direction of the applied pneumatic force.

PLPA can be used to perform curved profiles, can be manufactured with low cost, and can also be built with very long strokes. These characteristics, along with a favourable friction behaviour for servo control applications, make them very attractive in comparison with conventional pneumatic actuators. More details on the friction characteristics of PLPA can be found in [15]. However, PLPA suffers high mechanical stresses, as they are both compressed between the rollers and extended by the inner pressure. These mechanical constraints result in reduced endurance of the actuator, making them not suitable for industrial applications at their current stage of development.

In order to explore the causes and potential solutions for this drawback, we conducted initial investigations using conventional hoses. These studies showed that the hoses typically fail due to crack formation in regions where the hoses fold [14,18]. Regarding this issue, we developed hose geometries with material reinforcement at the folded areas and used additive manufacturing (AM) for the hose fabrication. Initial AM trials were conducted using stereolithography (SL) [14]. Investigations using stereolithography have shown that printed samples undergo crack formation almost immediately after the start of the experimental tests. As such, further trials were conducted using fused layer manufacturing (FLM) of thermoplastic polyurethane elastomer (TPE). Investigations using FLM of TPE (shore hardness of 82 A) have shown that geometries with geometrical reinforcement at the folded area can increase the average endurance of the hose more than twice when compared to a circular design [17]. Moreover, results of FLM trials of geometrical reinforced hoses yielded to a considerable increase in the number of life cycles (up to 50,000 cycles) [18]. However, 50,000 cycles still do not fulfil the industrial requirements for commercial use. As such, it would be necessary to use an alternative manufacturing technique which enables fabricating freeform geometries with longer life cycles.

As the next step, this study focused on the following points.

1. Effect of the 3D printing technique on the PLPA endurance: ARBURG Plastic Freeforming 3D printing technique was used for manufacturing of the hose prototypes. This technique was chosen as it uses the droplet injection method, which appears to yield in more isotropic prototypes when compared to the FLM technique.
2. Effect of the TPE material type (hardness) on the PLPA endurance: in this study, TPE materials with shore hardness levels of 60 A and 82 A were chosen for the printing material. TPE of 60 A is relatively more flexible when compared to 82 A. As such, circular hoses and the geometrically reinforced hoses were printed with both materials, and their corresponding endurances were tested in the PLPA setup.

To this end, several hoses with designs similar to the ones used in [17] were printed. Table 1 presents the designs that were tested in this work. It should be noted that the geometrically reinforced hose design was determined experimentally, keeping some geometrical constrains to ensure that the hose becomes fully flat when being compressed. Further details can be found in [17].

## 2. Experimental Setup

### 2.1. Material

3D printing experiments were conducted using two materials: material 1, TPE 60 A (Elastollan 1160 A P, supplied by BASF, Polyurethanes GmbH, Lemförde, Germany), and material 2, TPE 82 A (Filaflex 82 A, supplied by RECREUS, Alicante, Spain). The mechanical properties of the used materials are listed in Table 2

### 2.2. 3D Printer

**Figure 1:**
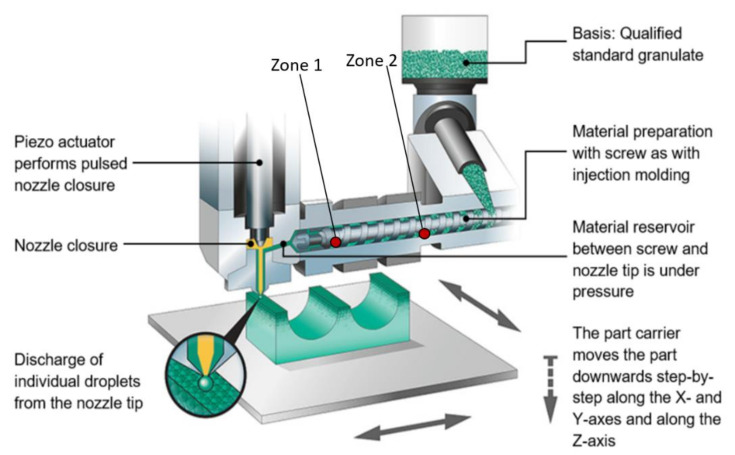
Printing principle of ARBURG Plastic Freeforming (APF) machine (Credit: Arburg GmbH + Co KG).

The experiments were conducted using ARBURG Plastic Freeforming (APF) technology with a freeformer 200-3X machine from ARBURG, Lossburg, Germany. The machine uses granulates as the raw material and plasticises them in a similar process to injection moulding. The molten polymer is discharged by the axial movement of the plasticising screw with a specific pressure. A nozzle closure system pulsed by a piezo actuator discharges up to 200 droplets of plastic per second with a diameter between 0.2 and 0.4 mm. Moreover, the precise positioning of the plastic droplets on previously calculated points is conducted by a movable three-axis build platform. This process is repeated in a layer-wise manner until the 3D object is manufactured. The machine has a build chamber volume of 154 mm × 134 mm × 230 mm. The build chamber can be heated up between 50 and 120 °C. The granulates are dried at 80 °C for 3–4 h before being guided to the print head. Pre-drying of the granulates avoids material processing problems that lead to bubble formation and thermal degradation due to moisture. A schematic view of the machine print principle is shown in Figure 1. In APF software, the following parameters could be varied in order to find the optimum printing parameters.

- Feed rate part carrier:
  ○ Speed of axes when moving from point to point without discharge of material.
- Feed rate, continuous extrusion:
  ○ Speed of the axes when discharging the filling of the part.
- Feed rate, discrete extrusion:
  ○ Maximum displacement speed of the axes when discharging the contour.
- Drop aspect ratio:
  ○ This parameter describes the ratio of the width to the height of a droplet after it has been discharged.
- Material discharge:
  ○ The material discharge is a nozzle flowrate parameter which determines the droplet volume.

It should be noted that the discrete extrusion is used for printing the outside perimeters (contours) where a high geometrical accuracy of the printed part is desired. The continuous extrusion is used to print the infill of the printed parts in order to increase the printing speed.

Initial trials were conducted for process parameter optimisation of TPE 60 A material. The first print attempts were performed using the available settings parameters of the APF software and material data sheet of TPE 60 A. In the design of the experiments, nozzle temperature and layer thickness were set to 200 °C and 0.2 mm, respectively. Further parameters such as build chamber temperature, extrusion speed, drop aspect ratio, and material discharge were varied. The examined parameters for the process optimisation of the TPE 60 A are listed in Table 3. Optimum process parameters were selected on the basis of visual inspection (naked eye and if necessary, also under the microscope) of the printed products. Inspections were mainly focused on the geometrical accuracy of the final hoses in such a way that the fabrication of bubble and crack free products is ensured.

Afterwards, optimised process parameters for TPE 60 A material were used for printing the TPE 82 A material. The printing results have shown that TPE 82 A can be successfully printed using the optimised process parameters of TPE 60 A material. It should be noted that the printing angle for all hose geometries were set to 90°. Printing without support structures resulted in nonhomogeneous structures within the top layers. This nonhomogeneous structure was caused by the vibration of the flexible layers during the print as they came in contact with the nozzle.

**Figure 2:**
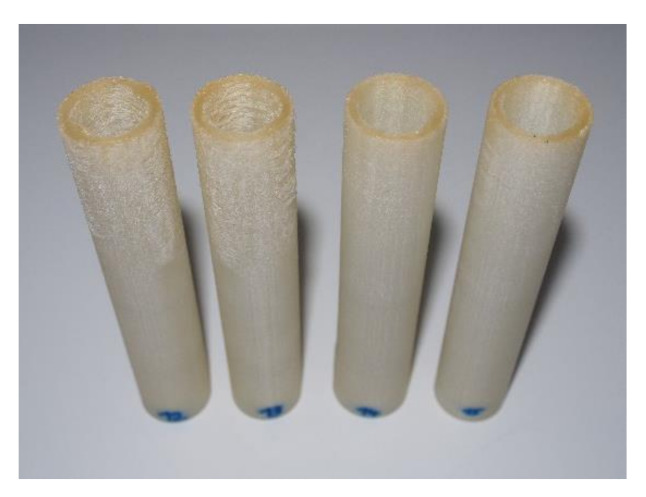
Progress in 3D printed part’s quality by using internal support structures from left to right.

As such, all hoses were printed with internal support structures in order to minimise the vibration of the printed hoses caused by their flexible nature. Figure 2 shows the progress in 3D printed part’s quality among the upper layers by using internal support structures, from left to right.

### 2.3. Pneumatic Test Bed

The use of double acting pneumatic actuators requires the existence of two independent chambers. In the case of linear peristaltic actuators, these chambers are formed by the compression of the hose between two rollers. To avoid leakages, which would negatively influence the energetic efficiency of the actuator, the rollers must tightly compress the hose. In order to control the compression force, we used adjustment screws connected to a spring, as detailed in [17]. The springs ensure that a nearly constant force is maintained during work, thereby ensuring that the leakage remains controlled, despite the fact that the hose walls become thinner due to the rollers’ compression [14]. Moreover, by measuring the displacement of the screws and knowing the spring stiffness, one can estimate the hose compression force, as is presented in Section 4.

**Figure 3:**
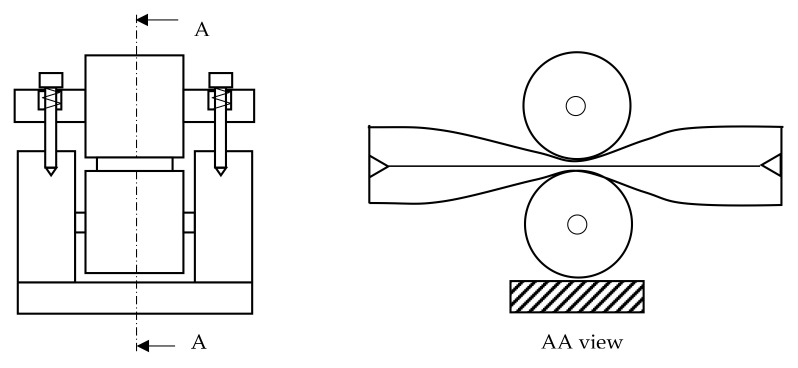
Mechanism to impose the force between rollers.

**Figure 4:**
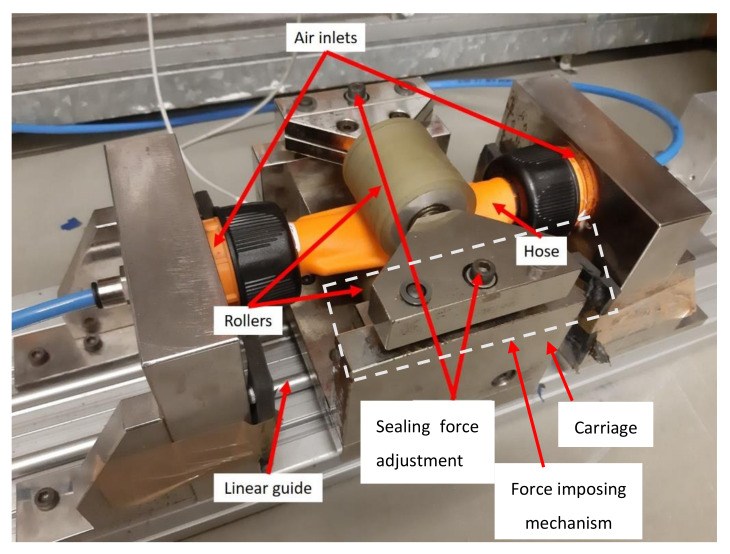
Experimental setup for leakage measurements.

Previous results in the literature showed that the configuration presented in Figure 3 leads to a considerable increase in the hose longevity [14] when compared to a configuration imposing the distance between rollers, and therefore in this work, the setup presented in Figure 4, which implements the principle depicted in Figure 3, has also been used.

The adjustment of the two spring washers was made in such a way that the leakage between chambers was lower than 5 slpm, regardless of the hose type. These leakages were determined using a mass flowmeter (Hastings HFM 201) and a pressure-reducing valve (Numatics Sentronic D), set for 3 bar (relative) working pressure. Further details can be found in [14].

The motion of the carriage was obtained by connecting the actuator (see Figure 4) to a 5/3 pneumatic directional valve, using the circuit described in detail in [14]. This circuit enables the actuator to move continuously between end positions.

## 3. Procedure

As mentioned in introduction, in this work, two hose designs were considered: designs A and B. Each design was printed using two different materials with different hardness levels. Material 1 is a soft and flexible (hardness 60 Shore A) material, while material 2 is a stiffer one (hardness 82 Shore A).

**Figure 5:**
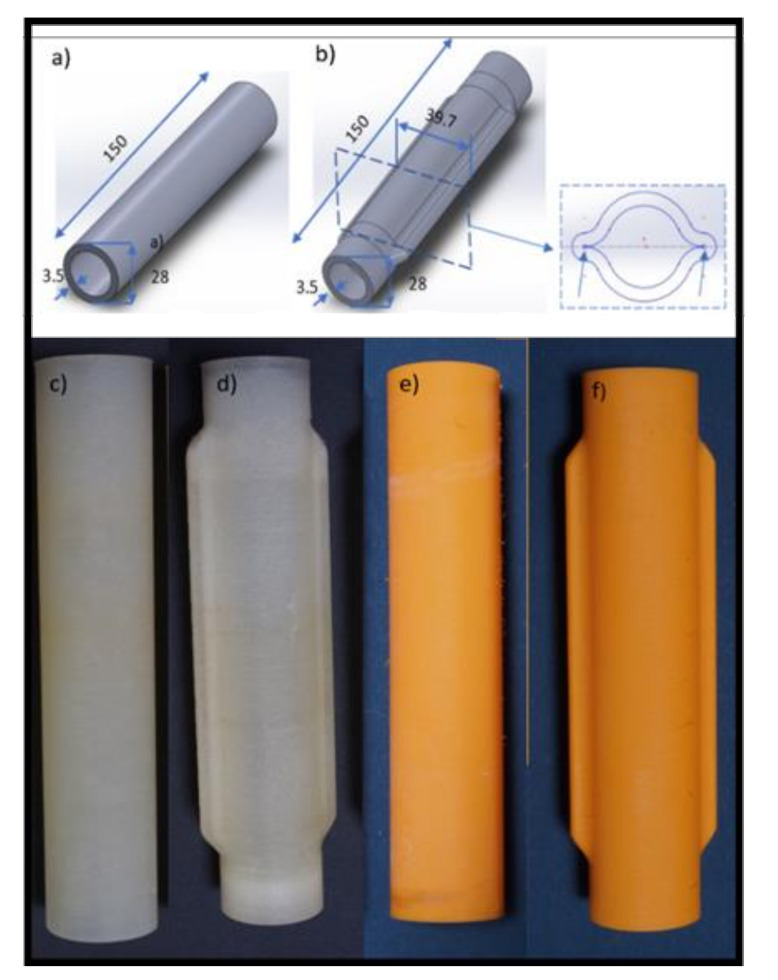
Geometrical details of the designs A and B (**a**,**b**) and pictures of the 3D printed parts for designs A1, B1, A2, and B2 (**c**–**f**), respectively. All dimensions are in millimeters.

Figure 5a,b present the detailed CAD models of these designs, while Figure 5c–f shows the corresponding 3D printed hoses. Designs A (circular cross section) and B (lip shaped cross section) were developed to demonstrate the influence of the hose design on the PLPA endurance. The underlying idea behind design B was to reinforce the bending areas while ensuring that, when the hose is compressed by the rollers, it becomes fully flat and sealed. This is an important aspect since it ensures that the pressure distribution is uniform, reducing possible leakage between chambers and thereby increasing the efficiency of the PLPA. In this entire manuscript, the circular printed hoses are mentioned as design A and the geometrically reinforced design is presented as design B. Printed hoses using TPE 60 A and TPE 82 A materials are mentioned as 1 and 2, respectively (see Figure 5).

Using the experimental setup presented in Section 2.3, we imposed a continuous back and forth motion of the actuator until the hose failure appeared. The stroke of the actuator setup was 60 mm. For each of the hoses presented in Figure 5, three samples were tested in the actuator setup with a constant source pressure of *P*_s_ = 3 bar. The leakages between the PLPA chambers were measured at the beginning of the experiments and during the trials, before the hose failed. No significant differences in these measurements were found.

## 4. Results and Discussion

**Figure 6:**
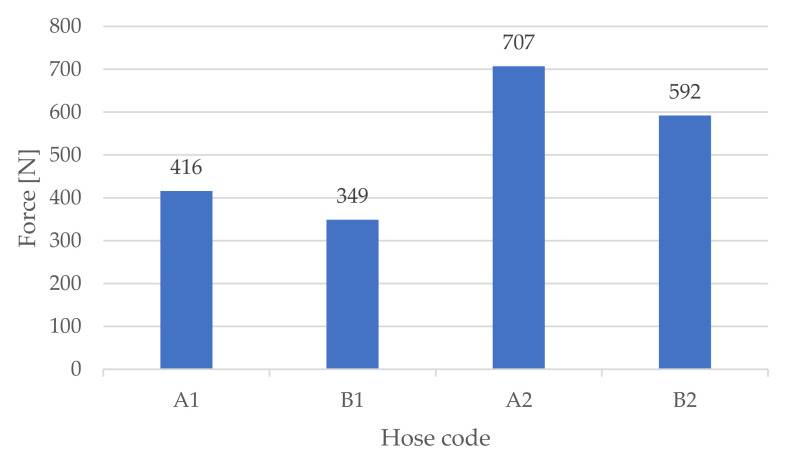
Hose compression force used for each hose design and material.

At the beginning of the trials, the hose compression force was estimated, as described in Section 2.3. The results are summarised in Figure 6.

**Figure 7:**
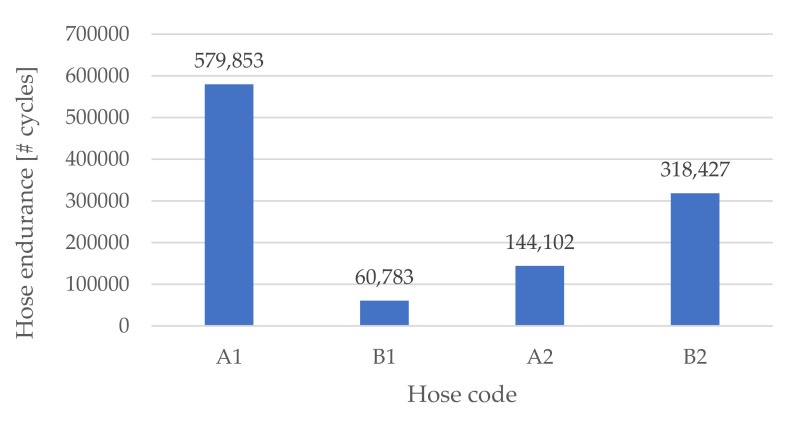
Average number of performed cycles for each hose until failure.

The PLPA endurance was tested for designs A and B and materials 1 and 2, as described in the previous sections. For each configuration—A1, A2, B1 and B2—three samples were tested. The average of the number of performed cycles for each hose is presented in Figure 7.

Analysing Figure 7, we were able to see that the overall obtained endurance in this work was considerably higher than the one obtained in previous works presented in the literature. Namely, when comparing the results presented in Figure 7 with the ones presented in [17], the highest average obtained endurance (579853 cycles for hose A1) was approximately 10 times higher than the one obtained in [17] (50247 cycles). Moreover, these endurance results are in the same order of magnitude as the ones obtained with commercial, fibre-reinforced hoses (about 320,000 cycles on average for the most durable hose [18]). Focusing on the average force values for each hose, we recognised that material 1 requires less clamping force than material 2. This suggests that a softer material needs to be subjected to less stress than a stiffer one to achieve a similar sealing effect. This is something expectable, given the lower stress at the same elongation of material 1 (see Table 2). The average required sealing force values were also higher for design A than for design B for materials 1 and 2. This shows that design B leads to less stress in the material while providing similar sealing. Another important conclusion is that the influence of the design on the endurance is highly dependent on the material type. In fact, although in previous studies [17] design B was shown to lead to a higher endurance than design A, in this study, the endurance results were dependent on the hose material. For the stiffer material 2 (TPE 82 A), this conclusion remained the same, while for the softer material 1, design A actually led to a higher endurance.

Two main mechanical stress causes are present in the normal working operation of a PLPA: (i) the stress caused by internal pressure and (ii) the stress caused by the compression force of to the rollers. In previous studies using conventional circular-shaped hoses, it was found that all tested hoses failed due to cracks on the hose folded edges. This suggests that cause (ii) is the predominant one when compared with cause (i). This was the reason why design B was developed, in order to reinforce the hose folded edges. Although design B has the potential geometrical reinforcement advantage, it also has the potential disadvantage of leading to localised stress at the folded areas [19], which might fragilise the hose with respect to cause (i). Given the different relative fatigue behaviour of materials A and B for similar designs, this study suggests that the appearing of localised stress as the predominant cause leading to failure is dependent on the material characteristics.

One possible justification to this fact is that softer materials tendentially lead to lower compression forces for similar sealing, as seen from the results presented in Figure 6, therefore potentially making cause (i) predominant. This suggests that design A is better suited for softer materials, while design B is better suited for stiffer materials.

**Figure 8:**
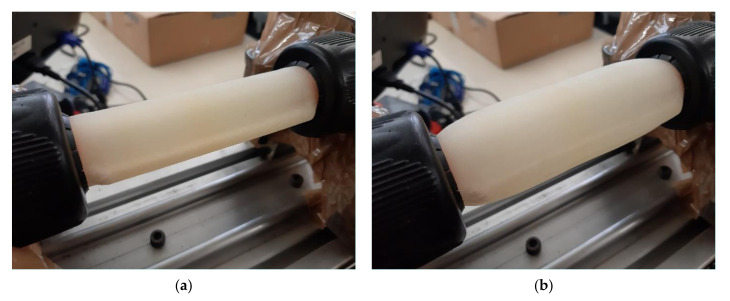

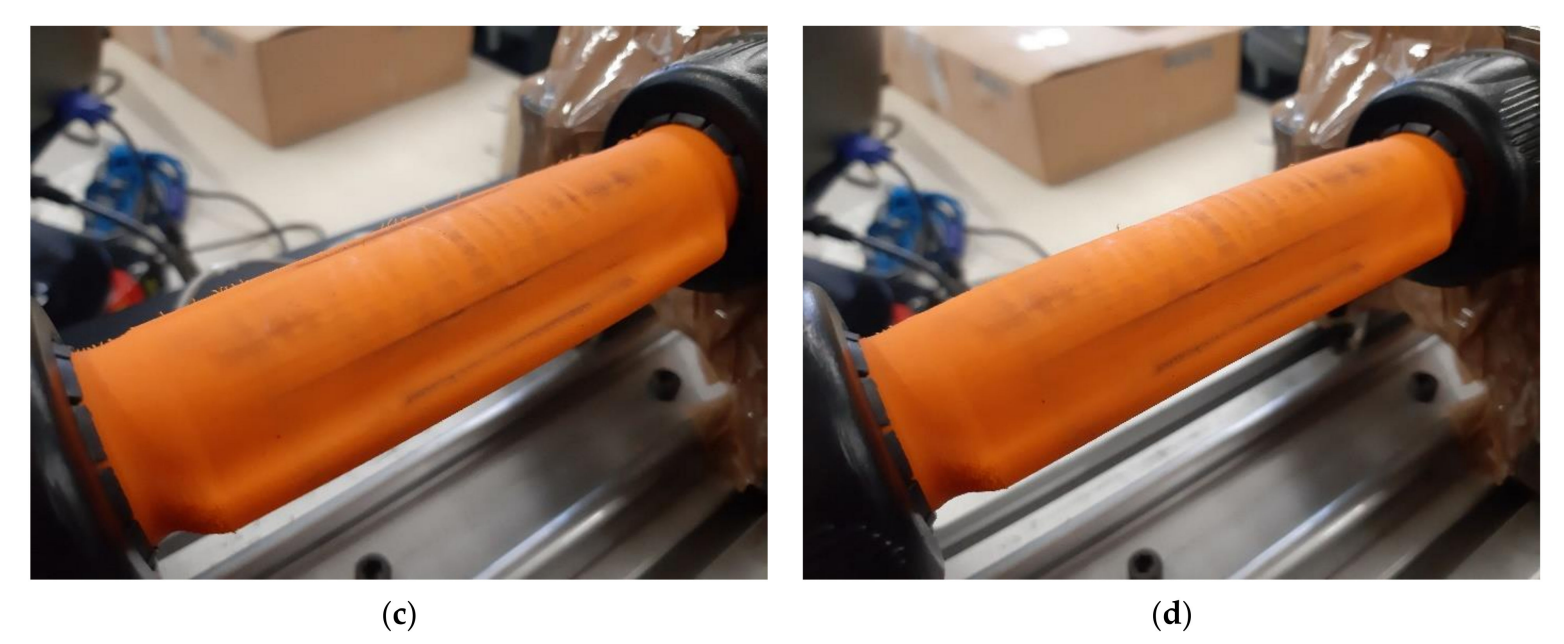
Influence of pressure (3 bar) in the hose shape: (**a**) B1 not pressurised; (**b**) B1 pressurised; (**c**) B2 not pressurised; (**d**) B2 pressurised.

Another possible justification to this lies in the fact that the benefits of design B might somehow be compromised with material 1 as the softer material 1 does not allow the hose to maintain its shape when being pressurised. In fact, with the stiffer material 2, design B hoses tend to maintain its shape when being pressurised, in contrast to the printed hoses with the softer material 1. To illustrate this phenomenon, Figure 8 presents a picture of hoses B1 and B2 not pressurised and under the influence of a 3 bar pressure. Clearly, hose B2 maintained its shape, while hose B1 tended to acquire a more “balloon-type” shape.

It can therefore be concluded that a compromise must be found between material and design in order to increase the hose longevity: in a softer material, effect (i) is predominant, and therefore design A might lead to higher endurance since it potentially leads to a more homogenous stress distribution. Furthermore, since the use of softer materials does not allow the hose to maintain design B shape, the benefits of this design might be compromised. In a stiffer material, effect (ii) appears to be predominant, and therefore design B with stiffer materials appears to lead to higher endurance. Further studies should therefore be made to explore materials with intermediate characteristics between materials A and B in an attempt to maximise longevity.

Finally, it should be underlined that the endurance results obtained by hoses printed using the ARBURG Plastic Freeforming technology are significantly higher than the ones obtained with the fused layer manufacturing technique [17]. This could be due to the fact that the droplet injection of ARBURG Plastic Freeforming technology leads to a more isotropic mechanical proprieties of the final part, similar to injection moulding. As such, it has been shown that the ARBURG Plastic Freeforming technique is more suitable for the prototyping of PLPA actuators.

## 5. Conclusions

In this study, several hoses for PLPA prototypes were printed using the ARBURG Plastic Freeforming technology. Two types of TPEs were used for printing different designs (conventional hoses with circular cross section and customised hoses using geometrical reinforcement at the folding areas). The 60 mm stroke hoses were experimentally tested for the 3D printed hoses in a PLPA setup. The experiments were conducted at 3 bar pressure, and the hose endurance was measured by running back and forth cycles.

Results showed that printed circular hoses using TPE 60 A (Elastollan 1160 A P, a TPE with 60 Shore A hardness) underwent four times more life cycles when being compared to the circular hoses printed using TPE 82 A (Filaflex 82 A, a TPE with 82 Shore A hardness). On the other hand, printed hoses with geometrical reinforcement at the folding areas using TPE 82 A underwent five times more life cycles when being compared to similar designed hoses printed using TPE 60 A. It can therefore be concluded that the influence of the hose design on its endurance is dependent on the type of hose material. In fact, printed hoses with more flexible materials require less clamping forces, therefore contributing to increasing the hose endurance, but also being more affected by designs that introduce stress concentration factors. Finally, the obtained PLPA endurance by printed hoses using ARBURG Plastic Freeforming technology are significantly higher when being compared to the results obtained with fused layer manufacturing technique. As such, it can be concluded that the ARBURG Plastic Freeforming technique is more suitable for prototyping of PLPA actuators. Future studies will focus on the use of a PLPA for water hydraulics as it has a lower ecological footprint.

## Figures and Tables

**Table 1:** Different views of the hose designs A and B.

Model	Design
A: Conventional hose design with circular cross-section	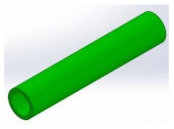
B: Geometrically reinforced hose design at the hoses folding areas	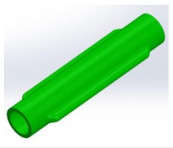

**Table 2:** Mechanical properties of the print materials.

Material	1	2
Shore hardness/(DIN ISO 7619-1)	60 A	82 A
Tensile strength (MPa)/(DIN 53504-S2/ISO 37)	27	45
Elongation at break (%)/(DIN 53504-S2/ISO 37)	750	600
Stress at 20% elongation (MPa)/(DIN 53504-S2/ISO 37)	1	2.5
Stress at 100% elongation (MPa)/(DIN 53504-S2/ISO 37)	2.5	6

**Table 3:** Process parameters of the TPE 60 A material.

Parameter	Range	Interval	Optimum
Build chamber temperature (°C)	0 and 60–100	20	60
Feed rate part carrier (mm/s)	100–250	50	200
Feed rate, discrete extrusion (mm/s)	10–20	15	5
Feed rate, continuous extrusion (mm/s)	40–65	5	50
Drop aspect ratio	1.28–1.36	0.02	1.30
Material discharge (%)	65, 67, 70	-	70
